# Quantification of Vitamins, Minerals, and Amino Acids in Black Walnut (*Juglans nigra*)

**DOI:** 10.3389/fnut.2022.936189

**Published:** 2022-07-27

**Authors:** Salma Akter Antora, Khanh-Van Ho, Chung-Ho Lin, Andrew L. Thomas, Sarah T. Lovell, Kiruba Krishnaswamy

**Affiliations:** ^1^Department of Biomedical, Biological and Chemical Engineering, University of Missouri, Columbia, MO, United States; ^2^Center for Agroforestry, School of Natural Resources, University of Missouri, Columbia, MO, United States; ^3^Department of Chemistry, University of Missouri, Columbia, MO, United States; ^4^Molecular Imaging and Theranostics Center, University of Missouri, Columbia, MO, United States; ^5^Department of Food Technology, Can Tho University, Can Tho, Vietnam; ^6^School of Natural Resources, University of Missouri, Columbia, MO, United States; ^7^Division of Plant Sciences, Southwest Research Center, University of Missouri, Columbia, MO, United States; ^8^Division of Food, Nutrition and Exercise Sciences, University of Missouri, Columbia, MO, United States

**Keywords:** agroforestry, cultivars, indigenous, metabolomics (OMICS), micronutrients, plant based food, food and health

## Abstract

This paper aims to quantify the micronutrients in black walnut and address its human health benefits. The metabolic profiling of 11 black walnut cultivars was accomplished using ultra-high-performance liquid chromatography coupled with quadrupole time-of-flight high-resolution mass spectrometer. Results revealed that the highest concentration of vitamin B_9_ was present in cultivar “Daniel” (avg. relative signal intensity 229.53 × 10^4^ mAU). “Surprise” and “Daniel” cultivars had the highest amount of vitamin B_5_. However, vitamin A, D_3_, E, and K showed no significant difference among the cultivars. The vitamin content levels among the cultivars were compared by applying one way ANOVA method with (*P* < 0.05) significance level. Mineral analysis for the black walnut kernel, Persian walnut, and black walnut protein powder was done using Inductively Coupled Plasma Optical Emission spectroscopy. The experimental data for black walnut kernel is 0.04 mg/g for Fe and 0.03 mg/g for Zn, and for black walnut, protein powder is 0.07 mg/g for Fe and 0.07 mg/g for Zn. The amino acid analysis and comparison with black walnut kernel show that black walnut flour and protein powder have a higher amount of essential and non-essential amino acids. Therefore, researchers, food process engineers, and food product developers should consider the health benefits of black walnuts and explore the commercial potential of this native agroforestry crop.

## Introduction

Black walnut (*Juglans nigra L*.) belongs to the family Juglandaceae and is indigenous to the Eastern and Midwestern United States (1). It is also known as Eastern black walnut or American walnut. It's the second-most-produced walnut species in the U.S., with Missouri leading the way (2). Because the wood is beautifully colored, sturdy, and easy to work with, black walnut is one of the most valuable North American hardwood timber species commercially and economically. The Persian walnut, sometimes known as the common or English walnut, is a closely related species (*Juglans regia* L.) indigenous to Central Asia and widely produced in most European countries.

**Figure 1:**
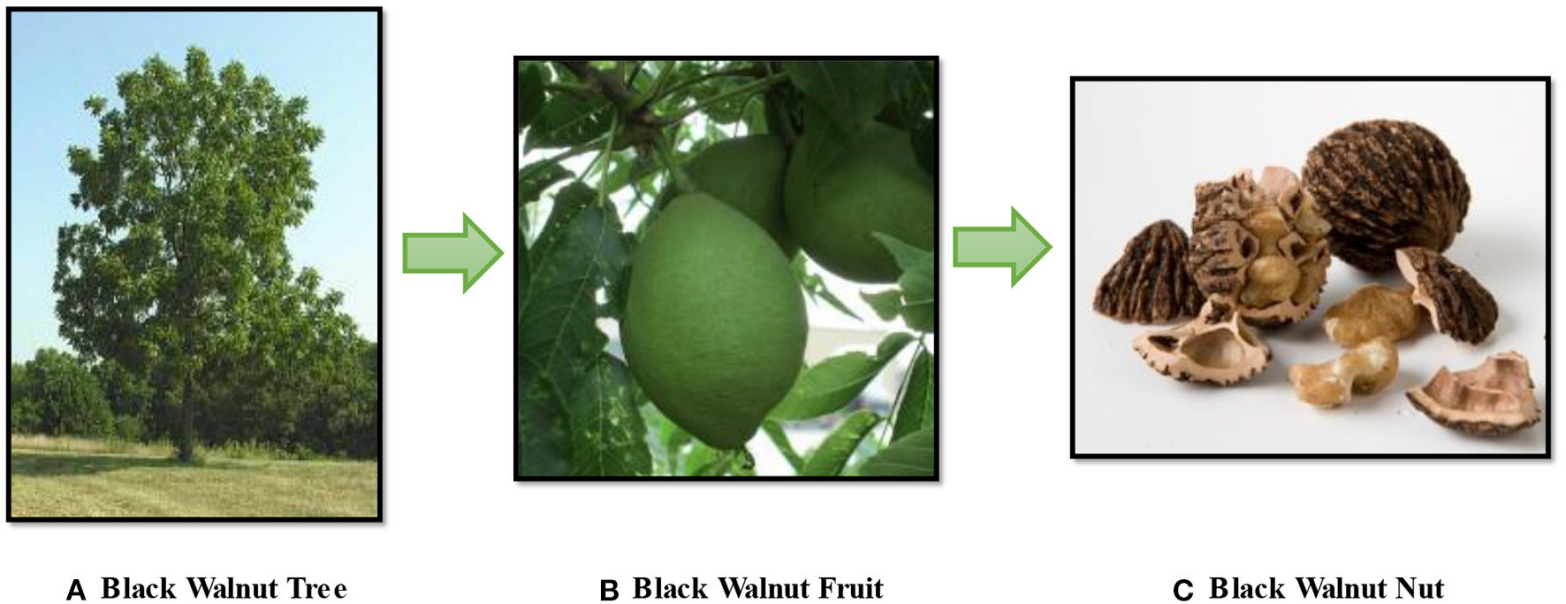
Black Walnut **(A)** Tree, **(B)** Fruit, **(C)** Nut (Photo Courtesy: Hammons Products Company, Stockton, Missouri).

The black walnut tree is a large, fast-growing tree that may attain 30–38 meters in height and 76–120 centimeters in diameter, and individual trees can live 100 years or more (3). The fruit consists of the pericarp or hull (fleshy outer cover), a very hard endocarp (shell), and the kernel (cotyledons and embryo) (Figure 1); the latter is commonly eaten raw or cooked. The shell of the black walnut is processed for use in a variety of industrial applications (4). The kernel is sweet, oily, and high in protein (4). Black walnuts are distinguished from other nut species by their distinct, robust flavor, incredible culinary diversity, and impressive health advantages. They are ideal ingredients for consumers concerned with food additives because they are all-natural product (5). Black walnut is one of the most beneficial and fully utilized native trees grown in natural forests in the United States.

While the health benefits of Persian walnuts, especially their vitamin and mineral profiles, have been well-documented (6), the indigenous black walnut has received little sporadic attention. Walnuts are known to be high in vitamin E and antioxidants (tocopherol) content, but black walnut contains a significantly greater amount of α-tocopherol and γ-tocopherol (a prevailing isomer) compared with Persian walnut (7, 8). A recent study by Zheng et al. (9) suggest that if the relationship and differences of different chemical components of walnuts are determined efficiently then it can be further applied in food industry (9). Although there is no significant research on the association of black walnut consumption with the health benefits of α-tocopherol and γ-tocopherol, recent studies have reported that these molecules are associated with preventing cancer and cardiovascular diseases (10).

Black and Persian walnuts have differing fatty acid, antioxidant, and amino acid profiles, according to USDA food and nutrition database statistics (8). According to recent studies, black walnuts contain protein, minerals, flavonoids, gallotannins, fatty acids, phytosterols, and phenolic compounds (2, 8, 11) although compared to Persian walnut, black walnut contains the same amount of phenolic compounds (12). The phenolic compounds available in black walnuts and tocopherol increase the antioxidant potential of this nut (2). The primary phenolic compounds available in black walnuts are tannins (13, 14), ellagic acid, gallic acid, ellagitannins, and hydrocinnamic acid. Compared with their commercially produced counterpart, Persian walnuts, the nutrient profile of black walnuts is promising. Black walnuts contain 24.06 g of protein per 100 g of kernels, which is ~15% of energy and substantially greater than Persian walnut (2). In addition, black walnuts are dense with dietary fiber, monounsaturated fatty acid (MUFA), and polyunsaturated fatty acid (PUFA), which is more than many other nuts (15). The PUFA (Linolenic acid and α- Linolenic acid) make up more than half of the total lipid content, which is significant given that these fatty acids are not produced in humans (16).

Black walnuts are a significant source of bioactive compounds with antioxidant and anti-inflammatory properties (17–19). Moreover, several black walnut cultivars possess antibacterial properties, which were identified through metabolomics analysis combined with bioassay-guided purification (11). The phenolic acids available in black walnuts, such as ferulic, p-coumaric, p-hydroxybenzoic, syringic, and vanillic acids, are beneficial to human health for their bioactivities (20). Syringic and vanillic acids found in Persian and black walnut have been reported to contribute to antioxidant activities (21, 22). Forty-nine phenolic compounds, which are well-known for their health benefits, were identified in black walnut kernels, with the types and levels of phenols differing significantly among cultivars studied (21).

This present study evaluates the concentration of vitamins among 11 black walnut cultivars using UHPLC coupled with QTOF high-resolution mass spectrometer. Liquid chromatography-tandem mass spectrometry (LC-MS/MS) analysis detected differences in these compounds' occurrence among 11 cultivars. The data from vitamin contents among the 11 cultivars were analyzed using ANOVA, and the difference between the means was analyzed using Tukey's honestly significance test (HSD). The amino acid profile of black walnut flour and black walnut protein powder were also determined. The mineral analysis of black walnut kernel and black walnut protein powder was evaluated following Schuster's 1988 method, and the data found is promising. Knowing the nutritional characteristics of various black walnut cultivars can provide more practical guidelines for health benefits and application in the food industry.

## Materials and Methods

### Black Walnut Collection

Samples of nuts from 11 black walnut cultivars were collected at the University of Missouri Horticulture and Agroforestry Research Center (39° 0'55 “N 92°45'5” W, New Franklin, MO, USA): Daniel, Davidson, Hay, Jackson, Kwik-Krop, Mystery, Shessler, Sparks 147, Sparrow, Surprise, and Tomboy. The black walnut trees were planted on soils that were well-drained and had a pH (6.5–7.2) that was close to neutral (23), and were well-managed for nut production with typical practices (e.g., pruning, fertilization, weed control, and pest and disease management) (24). The nut samples were collected in November 2017 upon maturity. They were mechanically de-hulled, dried at 24°C for 15 days, and then stored at −20°C until analysis.

Additionally, bulk, commercially produced black walnut kernels, flour, and protein powder samples were obtained from Hammons Products Co. (Stockton, MO) and stored in a freezer (−20°C). The black walnut protein powder is made from defatted black walnut kernels ground to a fine texture. Black walnut flour is made from unprocessed black walnut kernels ground to a fine texture. Persian walnut kernels from Natural Shelled Walnuts (California Walnuts, CA, USA) were purchased from the local market of Columbia, Missouri, USA.

### Sample Preparation

The 11 black walnut cultivar samples were prepared by extracting with methanol, as described in Ho et al. (11). First, the hulled nuts were gently split to extract the kernels from the shell. The kernels were then frozen at −20°C before being blended with a coffee grinder (Black + Decker, Beachwood, OH, USA, CBG100S). As described earlier, the kernels (10 g, 20–30 mesh) from each cultivar were recovered in 60 ml methanol for metabolomics analysis (11). The methanolic extract was sonicated for 60 min in a water bath (4°C), then centrifuged for 10 min at 8,000 rpm. The resultant supernatant was filtered *via* a syringe membrane filter (0.2 m, Whatman Anotop, Sigma-Aldrich, St. Louis, MO, USA) before being fed into high-resolution mass spectrometry using ultra-high-performance liquid chromatography (UHPLC-HRMS). Each cultivar was examined three times, with a methanol blank serving as a control.

### UHPLC-QTOF-MS Analysis

To measure the concentration of the major vitamins, UHPLC was used in conjunction with a maXis impact quadrupole time-of-flight (QTOF) high-resolution mass spectrometer (Bruker Co., Billerica, MA, USA) operating in both negative and positive electrospray ionization (ESI) modes with the nebulization gas pressure at 43.5 psi to analyze kernel extracts (2 l per injection) derived from 11 black walnut cultivars (11). Waters Acquit UHPLC ethylene bridged hybrid (BEH) C18 column (2.1 × 150 mm, 1.7 m particle size) maintained at 60°C was used to separate the black walnut samples. A mobile phase of 0.1% formic acid in water (A) and 100% acetonitrile was used (B). The gradient elution process began with a linear gradient of 95%: 5–30% eluents A: B in 30 min, followed by a linear wash gradient of 70–95% B in 30–33 min, 95% B in 33–35 min, 95–5% B in 35–36 min, and 5% B in 37–40 min at a flow rate of 0.56 ml/min. After data capture, mass spectrum data from 100 to 1,500 m/z were automatically gathered, three precursors were selected for auto MS/MS, and the m/z range was auto calibrated using sodium format.

### Data Processing

The UHPLC-MS data were analyzed based on the procedure described by Ho et al. (25) with some modifications. The LC-MS data obtained from UHPLC analysis were converted to NetCDF format files and then were processed by XCMS online platform (25)^1^. Multigroup analysis was performed with parameters optimized for UHPLC data as follows: a centWave method was used for feature detection (1 m/z = 10 ppm, minimum peak width = 5 s, and maximum peak width = 20 s); an obiwarp mode was selected for retention time correction (profStep = 1); chromatogram alignment was set as minfrac = 0.5, bw = 5, mzwid = 0.015, max = 100, minsamp = 1; adducts were optimized for UPLC/Bruker Q-TOF in both ESI(C) and ESI(–); and plant was selected for sample biosource for identification. Metabolites of significant features (intensity > 10,000) were selected for the annotation. These metabolites, with the accurate mass of the molecular ions (m/z values) that match with the m/z values of vitamin, referenced to METLIN metabolite^2^ mass spectral database containing over 1 million molecules, were used to determine vitamin metabolic profiles in each cultivar (26).

### Statistical Analysis

Standard Microsoft Excel ^TM^ spreadsheet was used to determine whether the vitamin levels among the 11 cultivars were statistically different from each other. Data were reported as mean ± standard deviation (S.D.), and the average intensity values of the vitamins among cultivars were compared by applying a one-way ANOVA test with a significance level of *P* < 0.05. The differences between means were determined using Tukey's honestly significant difference test using JMP 14.0 software: SAS Institute Inc., Cary, NC.

### Mineral Analysis Using ICP-OES

Black walnut kernel and Persian walnut kernel were analyzed for minerals using Inductively Coupled Plasma Optical Emission spectroscopy (ICP-OES). ICP-OES is a widely used multielement technique with a large linear dynamic range, excellent analytical sensitivity, and high sample throughput (27, 28). The model Varian Vista-MPX CCD simultaneous ICP-OES was used for this analysis. Microwave digestion was used to prepare the kernel samples for total metal analysis of Zn, Fe, Mn, Cu, K, Ca, Mg and P. For 0.5 g of sample, 10 μl concentrated HNO_3_ was added. A preloaded method for the MARS microwave digestion system (CEM Corporation, USA) was used to digest the sample. Once cooled, the solution was diluted to 50 ml using deionized water (DI water). To determine total nitrogen (N), the combustion method was used by the Elementar CNS. The mineral analysis was conducted at the Soil and Plant Testing Laboratory, Columbia, University of Missouri. The mineral analysis of black walnut protein powder was performed by Eurofins Microbiology Laboratories (Des Moines, Iowa) (Lab-2), where sample preparation and analysis were conducted following AOAC 984.27, 927.02, 985.01, 965.17 mod, respectively (29).

### Amino Acid Analysis

The amino acid data of black walnut kernel and black walnut protein powder were collected from two companies, both using HPLC: Intertek Agricultural Services, New Orleans, LA (Lab-1), and Eurofins Microbiology Laboratories (Des Moines, Iowa) (Lab-2). All the samples were prepared, and values were determined using TAALC_S described by 28 except for tryptophan which was determined using the TRPLC_S method (30). The amino acid composition was reported as mg amino acid per g of protein.

## Results and Discussion

### Macronutrient Profile of Black Walnut

#### Vitamins

**Figure 2:**
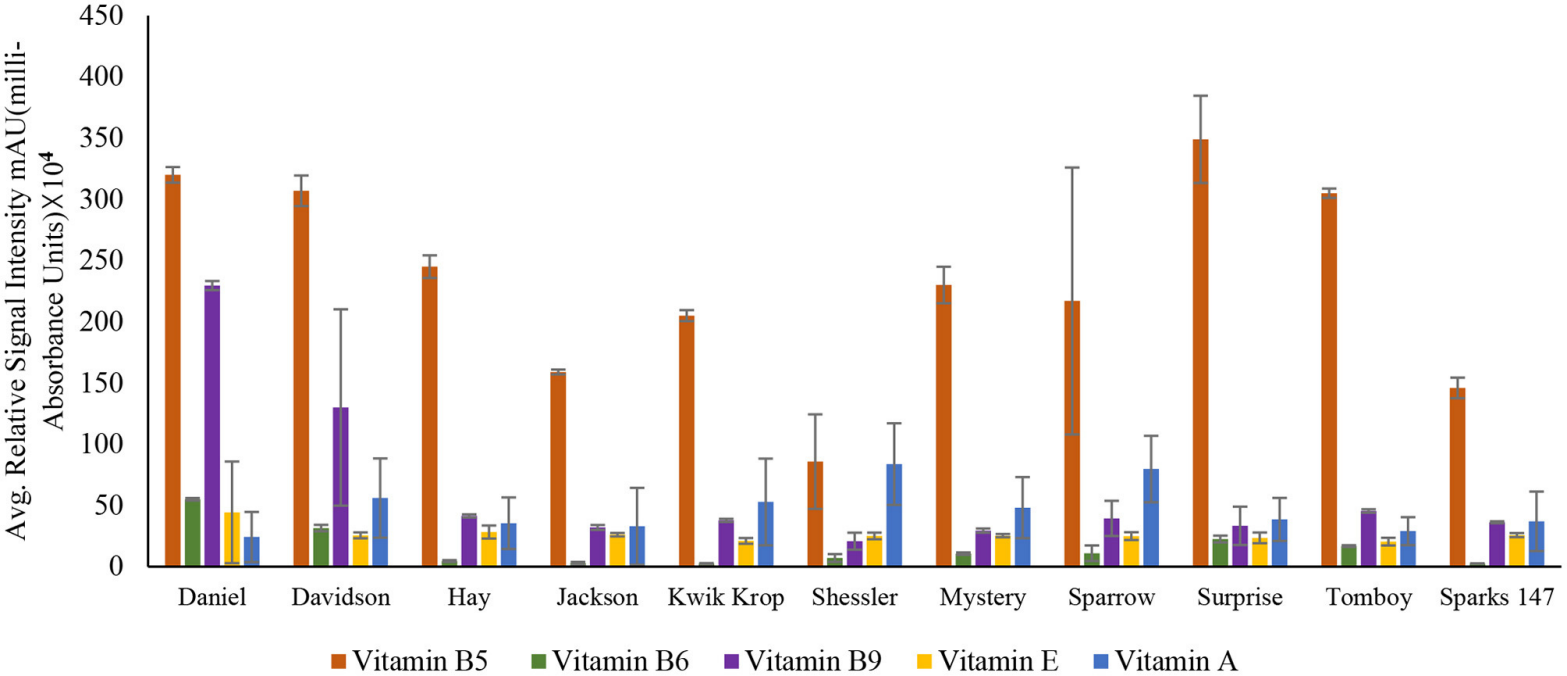
Concentration of vitamin B_5_ (Pantothenic acid), vitamin B_6_ (Pyridoxine), vitamin B_9_ (Folic acid), vitamin E (Tocopherol), and vitamin A (Retinol) in 11 Black Walnut cultivars.

**Figure 3:**
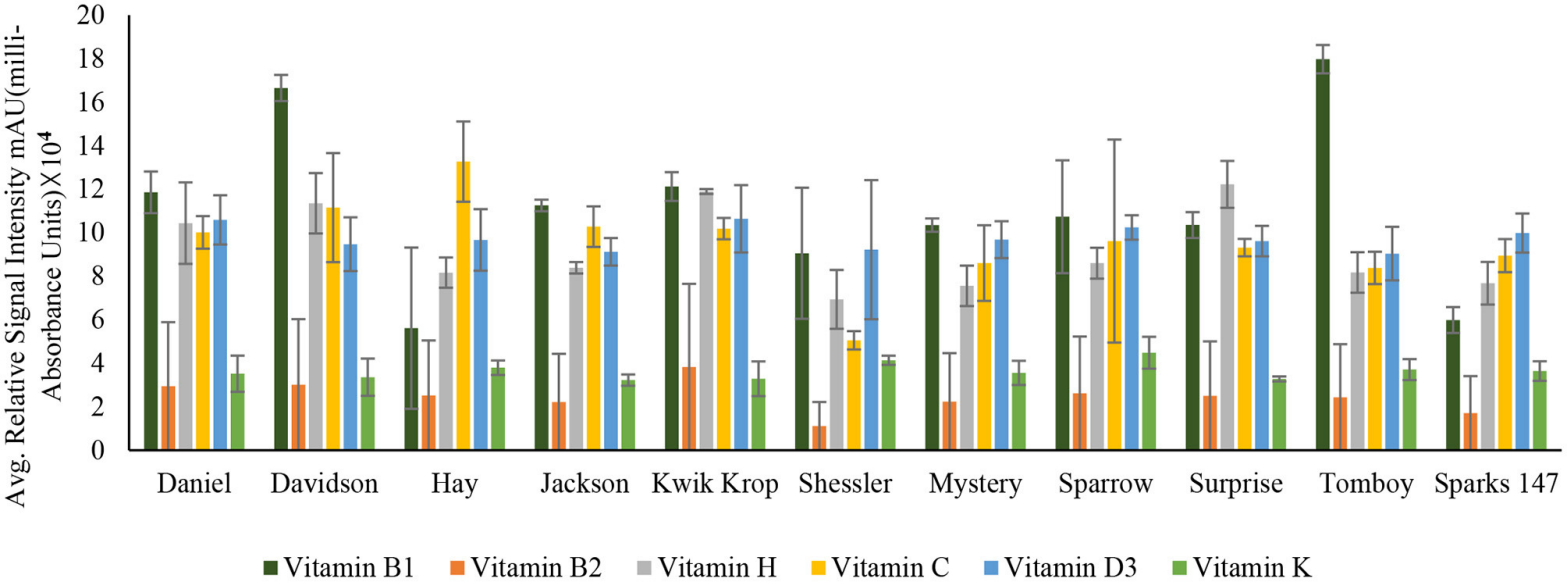
Concentration of Vitamin B_1_ (Thiamin), Vitamin B_2_ (Riboflavin), Vitamin H (Biotin), Vitamin C (Ascorbic acid), Vitamin D_3_ (Cholecalciferol) and Vitamin K (Phylloquinone) in 11 black walnut cultivars.

Among all the vitamins detected in the 11 black walnut cultivars shown in Figures 2, 3 (refer Supplementary Table S1), all cultivars had numerically the largest amount of vitamin B_5_. Vitamin B_5_, also known as pantothenic acid, is a necessary component for human health. Pantothenic acid deficiency can cause numbness and burning feeling in the hand and feet, a decrease in antibody production, insomnia, and fatigue (31).

The results indicated that for vitamins B_1_, B_2_, B_5_, B_6_, B_9_, H, and C, there were significant differences (*P* < 0.05) in vitamin content among the cultivars, likely due to genetic variation. Vitamin B_1_ (thiamin) and B_2_ (riboflavin) are essential nutrients for the body's correct blood function (32) and immune system, as well as good skin and hair (33). Other vitamins, such as pyridoxine and folate, require B_2_ for their metabolism (33, 34). The average amount of Vitamin H and C found in all 11 cultivars was 9.2 ± 0.94 and 9.5 ± 1.39, respectively. Vitamin H (biotin) is mainly required for protein and keratin synthesis, thereby playing an essential role in maintaining healthy hair and nail growth in the human body (35). Compared to Persian walnut, Black walnuts are higher in Vitamin C concentration (8). Vitamin C, commonly known as ascorbic acid, is an antioxidant required to produce certain amino acids. It also acts as a skin barrier and has anti-aging properties (36).

For vitamins B_6_ and B_9_, the cultivars Daniel and Davidson had statistically higher levels compared with other cultivars. The concentration of vitamin B_6_ in cultivar Daniel and Davidson is 55.00 ± 1.06 and 31.5 ± 2.60 mAU, respectively. Vitamin B_6_ (pyridoxine) is the most functional coenzyme that helps in the cognitive development of neurotransmitter synthesis, immune function, gene expression, and hemoglobin formation (37, 38). The concentration of vitamin B_9_ found in Daniel and Davidson is 229.53 ± 3.72 and 130.01 ± 80.20 mAU, respectively. Vitamin B_9_, or folic acid, is required for cognitive function, cardiovascular diseases, cancer DNA synthesis, and gene expression (39). For vitamins A, D_3_, E, and K, the significance level was >0.05, suggesting that they are statistically similar amongst the cultivars and may not be influenced by genetics. However, Supplementary Table S1 shows the average amount of vitamin A found in all the 11 cultivars is 47.1 ± 25.39.

Vitamin A (retinol) insufficiency can lead to major health problems; it is estimated that 30% of children under the age of 5 are vitamin deficient and vitamin A deficiency accounts for 2% of all deaths in specific age groups (36). The average amount of vitamin D_3_ found in all black walnut cultivars was 9.7 ± 1.22 mAU. Vitamin D_3_ or cholecalciferol is necessary for the regulation of physical functions in the human body, especially required for bone health (40). The large amount of γ- tocopherol (vitamin E) available in black walnuts compared to Persian Walnut (8) associated with protection against prostate cancer (41). γ- Tocopherol, for example, inhibited the proliferation of tumor cells and cancer cells, including prostate cancer cells (42), also known to prevent the effects of cardiovascular diseases (20). Vitamin K or Phylloquinone was found in minimal amounts in all the cultivars. However, vitamin K insufficiency can be life-threatening since it can cause excessive bleeding and is also linked to osteoporosis and cancer (43).

#### Mineral Analysis

**Figure 4:**
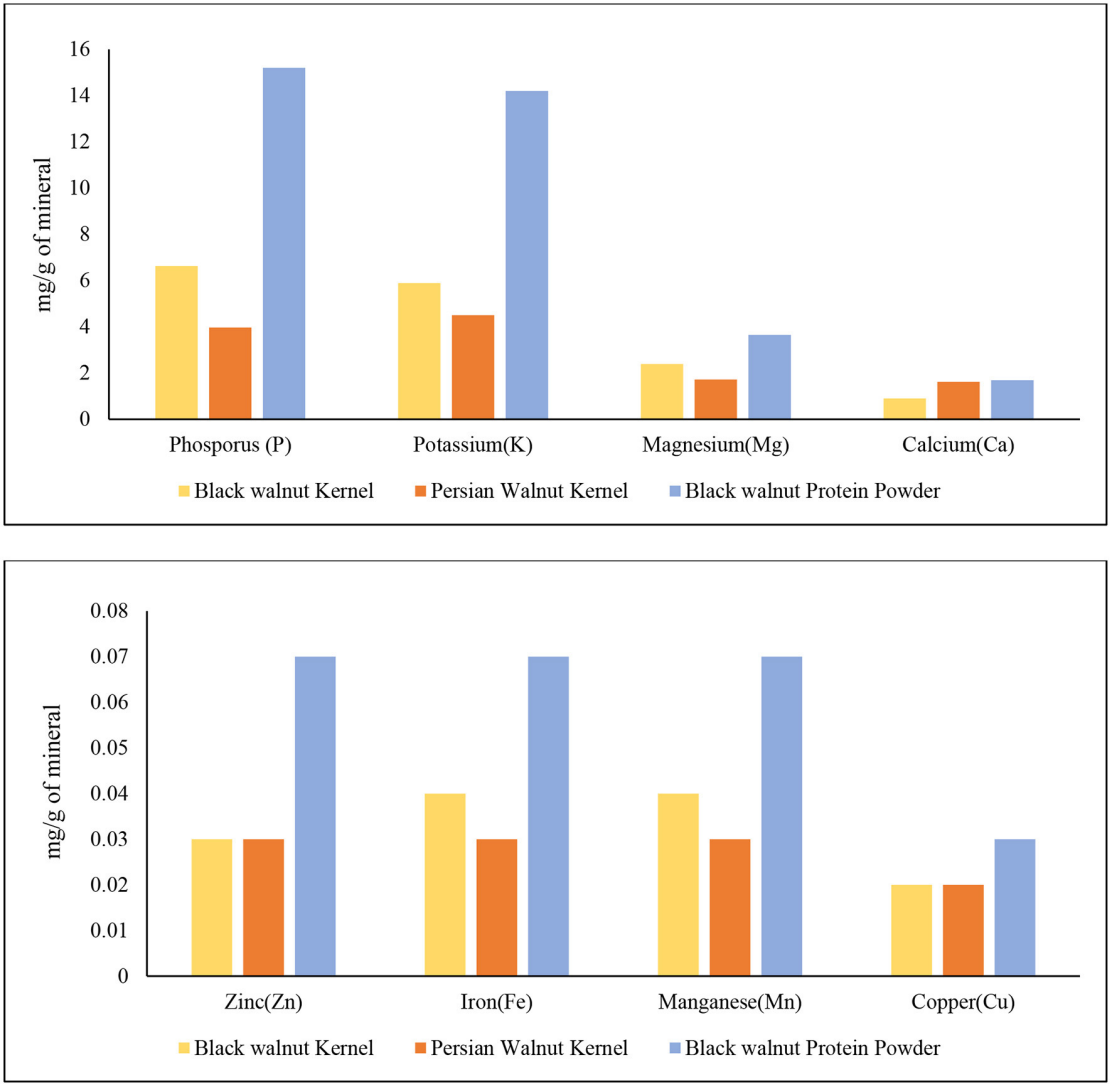
Mineral analysis of Black walnut kernel, Persian walnut, and Black walnut protein powder (Lab-2).

The mineral analysis of black walnut kernel and black walnut protein powder obtained from Hammons shows promising results compared to Persian walnut. Figure 4 demonstrates that the black walnut kernel and black walnut protein powder contain high amounts of N, P, and K content compared to Persian walnut. The sequence is determined by the contents (mg/g) of black walnut kernels was N>P>K>Mg>Ca>Mn>Fe>Zn>Cu, while the order in Persian walnut was N>K>P>Mg>Ca>Zn>Mn>Fe>Cu, and for black walnut, protein powder was N>P>K>Mg>Ca>Zn>Mn>Fe>Cu. The data published by USDA (34) and the data reported show similarity among minerals Ca, Fe, Mg, P, K, Zn, Mn, and Cu for both black and Persian walnut kernels.

Nitrogen is necessary for all life because it is the building block of all amino acids. Malfunctions in the ubiquitin protein degradation pathway are one of the most common disorders linked with nitrogen metabolism. This route is essential for maintaining or destroying the correct proteins within a cell (44). Black walnut protein powder contains higher amount of nitrogen which is 96.6 mg/g compared to black walnut kernel 43.5 mg/g and Persian walnut 27.6 mg/g, respectively. Phosphorus plays a vital role in the human body by helping in bone mineralization, signal transduction, and ATP synthesis (45). Phosphorus deficiency can cause bone loss (46, 47), myopathy (48), and frailty (45). The mineral profile of black walnut kernel and black protein powder shows that the Phosphorus content was higher for the protein powder (15.2 mg/g) and black walnut kernel (6.63 mg/g) compared to Persian walnut, which was 3.97 (mg/g). Figure 4 also shows that potassium is the third-highest mineral found in black walnuts, which is also higher than in Persian walnuts. Potassium supplementation has been demonstrated to improve human health, especially in terms of cardiovascular diseases, in epidemiological research, animal experiments, and therapy trials (49).

Magnesium deficiency has been associated with osteoporosis, hypertension, coronary heart disease, congestive heart failure, arrhythmia, diabetes mellitus, asthma, migraine headaches, and pre-eclampsia (50). In Figure 4, both black walnut kernel and black walnut protein powder had Mg in the highest amount. Then there's Ca, which is recognized to be essential for bone and tooth formation and health. Furthermore, Ca serves as a vital electrolyte for the human body (51). Although black walnut kernel and protein powder had a numerically higher amount of Mn, Zn, Fe, and Cu than Persian walnut, the difference was minimal. In the human body, zinc is one of the most prevalent nutritionally necessary minerals. Muscle and bone contain 85% of total Zn, skin, and liver contain 11%, and all other tissues contain the remaining 4% (52). Mn is related to the metabolism process in the human body and is important in the neurological and heart function process (53). Fe is known to be an essential micronutrient for good health and normal functioning of the body. Cu serves as a cofactor for a various enzyme in the human body (52, 54). It is a dynamic element that has anti-inflammatory, antiviral properties.

Black walnuts have robust, bold taste compared to Persian walnut (55), this may be due to high potassium, and phosphorus content as potassium has a salty, bitter taste. However, it is also reported that the color of the black walnut kernel can be affected by delayed harvesting and hulling which may lead to unpleasant flavor (56). Thus, further research on volatile and mineral compounds linked to flavor of black walnuts should be conducted to understand the quality, shelf-life, nature, and develop value added nutritional products of this nut.

#### Amino Acid Content

**Figure 5:**
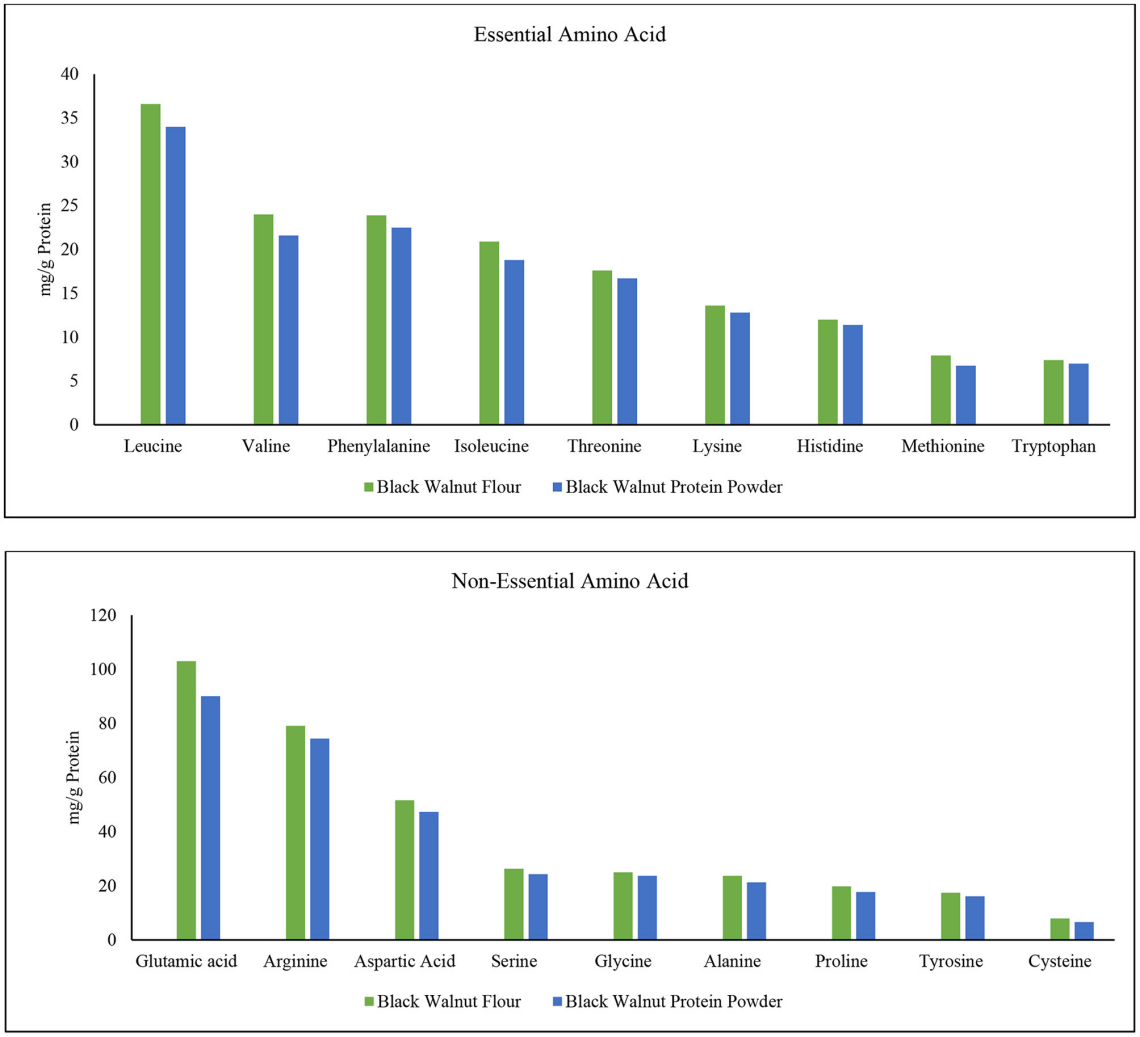
Essential and non-essential amino acid composition in black walnut flour (Lab-1) and black walnut protein powder (Lab-2).

The amino acids phenylalanine, valine, threonine, tryptophan, isoleucine, methionine, leucine, lysine, and histidine are critical for human life (57). These are essential amino acids which the human body cannot produce. Since humans do not manufacture essential amino acids, they serve a critical function in body development. Conditionally essential amino acids are arginine, cysteine, glycine, glutamine, histidine, proline, serine, and tyrosine (58, 59). Four different varieties of walnut kernels are said to have 16 different amino acids (60), and among them, leucine was found in highest amounts. Figure 5 shows that leucine content is highest among all the amino acids in black walnut. Moreover, leucine was comparatively higher in black walnut flour than in protein powder. In terms of protein synthesis and degradation, leptin secretion, energy balance modulation, and so on, leucine appears to be the most potent (61). The order depending on the content of essential amino acid (mg/g) in black walnut flour is leucine> valine> phenylalanine> isoleucine> threonine> lysine> histidine> methionine> tryptophan, while the order in black walnut protein powder was leucine> phenylalanine> valine> isoleucine> threonine> lysine> histidine> tryptophan> methionine. The second highest content is valine which is comparatively higher in black walnut flour than in protein powder. The essential acid methionine is found to be the lowest amount in black walnut flour than in protein powder, but the difference is negligible.

Unlike essential amino acids, a healthy human body could produce non-essential amino acids if food enriched with protein is taken on a daily dietary basis. Non-essential amino acids, on the other hand, help with tissue growth and repair, immunological function, hormone production, and the formation of red blood cells in the human body (34). The order depending on the content of non-essential amino acid (mg/g of protein) in black walnut flour is glutamic acid> arginine> aspartic acid> serine> glycine> alanine> proline> tyrosine> cysteine, while the order in black walnut protein powder was glutamic acid> arginine> aspartic acid> serine> glycine> alanine> proline> tyrosine> cysteine.

In general, plant-based protein contains less calories and fat than animal-based protein while being high in fiber and essential nutrients, making these protein sources a good alternative for healthy diets. As a plant-based edible product, black walnuts are a rich source of protein, containing a low amount of sugar compared to Persian walnut (8). The edible nut rich in bioactive and other compounds could be a source of protein for maintaining a healthy diet. For instance, Chickpea and pea contain 20.5 g and 5.42 g of protein in a 100g portion (34) respectively, which is low compared to black walnut. The amount of sugar in black walnut is 1.1g/ 100g, which is low compared to chickpea and pea at 10.7g and 5.67g in 100g portion, respectively (34). Black walnut protein powder contains a high level of mineral content which could also be incorporated into a daily diet to promote health benefits.

## Conclusion

Black walnuts are prized for the kernels' bold, distinctive, earthy flavor and fine-quality hardwood timber. This study demonstrated that vitamin analysis of 11 black walnut cultivars, vitamin B_1_, B_2_, B_5_, B_6_, B_9_, H, and C, there were significantly different in vitamin content among the cultivars, but vitamin A, D_3_, E, and K showed no significant difference among the cultivars. The significant differences in vitamin content among black walnut cultivars in this study suggest a genetic basis for these important nutrients, which could likely be further improved *via* breeding.

The mineral profile of black walnut kernel, Persian walnut, and black walnut protein powder also shows that N, P, and K are available in high concentrations compared to other micronutrients. Moreover, the amount of Zn, Fe, Mn, and Cu are almost similar in the Black walnut kernel, Persian walnut, and black walnut protein powder. Amino acid analysis among black walnut flour and black walnut protein powder show that black walnut flour contains a higher amount of essential and non-essential amino acids than black walnut protein powder.

Black walnuts are also dense with high nutrient compounds, including lipids, protein, fiber, vitamins, minerals, and bioactive molecules such as phenolic compounds. However, the current market value of black walnut kernels is not as high as Persian walnut due to unfamiliarity. In addition, the distinct flavor makes this nut less likely to be used as a snacking or table nut. By determining the potential health-promoting micronutrients (vitamins and minerals) present in black walnuts, the case for its economic production and increased marketing might be strengthened. The excellent vitamin and mineral contents increase the potential for black walnuts in the competitive market. The reported micronutrient analysis shows that the kernel and some kernel products such as flour and protein powder contain a high level of bioactive compounds (vitamin B_9_, B_5_, A, Fe, Zn) required to support human health. Despite the lack of research into the capacity of black walnut to reduce disease, based on the reported data, it can be assumed that consumption of black walnut could contribute to protecting humans against numerous diseases, including diabetes, neurodegenerative, cardiovascular diseases, and cancer.

Moreover, black walnuts are used in different recipes like cake, ice cream, salad, and desserts to increase the food's palatability and flavor. In summary, black walnuts have a rich micronutrient profile, offering great potential to promote human health compared to other commercial nuts available on the market. This research will provide recommendations for the usage of black walnut resources for researchers as well as new notion for future research. In addition, a greater understanding of the health benefits of black walnut and the preservation of bioactive compounds should be the focus of future research and clinical investigations to support human health effectively.

## Data Availability Statement

The raw data supporting the conclusions of this article will be made available by the authors, without undue reservation.

## Author Contributions

SA: writing—original draft, formal analysis, investigation, data curation, and visualization. K-VH: formal analysis, investigation, and writing—review and editing. C-HL: formal analysis, resources, investigation, and writing—review and editing. AT: writing, editing, and contextualizing the work. SL: writing—review and editing and funding acquisition. KK: conceptualization, supervision, formal analysis, resources, writing—review and editing, project administration, and fund acquisition. All authors contributed to the article and approved the submitted version.

## Funding

This work was supported by USDA Hatch Funds (MO-HAFE0003) Food Engineering and Sustainable Technologies (FEAST), University of Missouri, the University of Missouri Center for Agroforestry, and the USDA/ARS Dale Bumpers Small Farm Research Center, Agreement number 58-6020-0-007 from the USDA Agricultural Research Service.

## Conflict of Interest

The authors declare that the research was conducted in the absence of any commercial or financial relationships that could be construed as a potential conflict of interest.

## Publisher's Note

All claims expressed in this article are solely those of the authors and do not necessarily represent those of their affiliated organizations, or those of the publisher, the editors and the reviewers. Any product that may be evaluated in this article, or claim that may be made by its manufacturer, is not guaranteed or endorsed by the publisher.

## Footnotes

^1^ www.xcmsonline.scripps.edu

^2^ http://metlin.scripps.edu

## Abbreviations

ANOVA: analysis of variance
BEH: ethylene bridged hybrid
CHD: coronary heart diseases
DI water: deionized water
ESI: electrospray mass spectrometer
ICP-OES: Inductively Coupled Plasma Optical Emission spectroscopy
I.L.: interleukin
LDL: low density lipoprotein
mAU: milli Absorbance Unit
MCP: monocyte chemoattractant protein
MI: myocardial infection
QTOF-HRMS: Quadrupole Time-of-flight high resolution mass spectrometer
UHPLC: ultra-high-performance liquid chromatography
UHPLC-HRMS: ultra-high-performance liquid chromatography high-resolution mass spectrometry.
