# Supplementary material for: Quantification of Vitamins, Minerals, and Amino Acids in Black Walnut (Juglans nigra)
Source: Front Nutr. 2022 Jul 27;9:936189. doi: 10.3389/fnut.2022.936189 (PMC9363771; doi:10.3389/fnut.2022.936189)
Supplement: Supplementary file 1 [file Table_1.docx]

**Supplementary Data:**

**Table S1:** Statistical analysis (one-way ANOVA results) of vitamins among the 11 cultivars of black walnut (Mean value of individual vitamins/ cultivar provided) (All values are raised to the 4^th^ power of 10)

| **Cultivars** | **Vitamin B_1_, mAU** | **vitamin B_2_, mAU** | **Vitamin B_5_,**  **mAU** | **Vitamin B_6_, mAU** | **Vitamin B_9_, mAU** | **Vitamin**  **H, mAU** | **Vitamin**  **C, mAU** | **Vitamin**  **A, mAU** | **Vitamin D_3_, mAU** | **Vitamin**  **E, mAU** | **Vitamin K, mAU** |
| --- | --- | --- | --- | --- | --- | --- | --- | --- | --- | --- | --- |
| **Daniel** | 11.9±0.9^bc^ | 2.9±0.42^ab^ | 320.0±6.38^ab^ | 55.0±1.06^a^ | 229.5±3.72^a^ | 10.4±1.87^abc^ | 10.0±0.75^ab^ | 24.2±20.49^a^ | 10.6±1.13^a^ | 44.4±41.50^a^ | 3.5±0.83^a^ |
| **Davidson** | 16.7±0.60^ab^ | 3.0±0.47^ab^ | 307.0±12.50^abc^ | 31.5±2.60^b^ | 130.0±80.20^b^ | 11.4±1.39^ab^ | 11.2±2.51^a^ | 56.0±32.38^a^ | 9.5±1.24^a^ | 25.6±2.39^a^ | 3.4±0.85^a^ |
| **Hay** | 5.6±3.71^e^ | 2.5±0.21^abc^ | 245.0±9.24^abcd^ | 4.6±0.77^ef^ | 41.4±1.27^c^ | 8.2±0.70^cd^ | 13.3±1.84^a^ | 35.5±21.05^a^ | 9.7±1.42^a^ | 28.2±5.32^a^ | 3.8±0.33^a^ |
| **Jackson** | 11.2±0.27^c^ | 2.2±0.52^bc^ | 159.0±2.00^de^ | 2.8±1.06^f^ | 32.0±2.00^c^ | 8.4±0.26^bcd^ | 10.3±0.93^ab^ | 33.0±31.35^a^ | 9.1±0.63^a^ | 26.1±1.44^a^ | 3.2±0.26^a^ |
| **Kwik-Krop** | 12.1±0.66^bc^ | 3.8±0.85^a^ | 205.0±4.52^cd^ | 2.5±0.34^f^ | 37.8±1.31^c^ | 11.9±0.11^a^ | 10.2±0.49^ab^ | 52.9±35.37^a^ | 10.6±1.55^a^ | 21.1±2.32^a^ | 3.3±0.80^a^ |
| **Shessler** | 9.1±3.01^cde^ | 1.1±0.34^c^ | 85.8±38.60^e^ | 7.1±3.18^ef^ | 20.8±6.94^c^ | 6.9±1.35^d^ | 5.1±0.42^b^ | 83.8±33.35^a^ | 9.2±3.20^a^ | 25.1±2.71^a^ | 4.1±0.21^a^ |
| **Mystery** | 10.4±0.31^cde^ | 2.2±0.26^bc^ | 230.0±14.90^bcd^ | 10.7±0.98^de^ | 29.4±1.69^c^ | 7.6±0.93^cd^ | 8.6±1.74^ab^ | 48.2±24.91^a^ | 9.7+0.85^a^ | 25.3±1.31^a^ | 3.6±0.56^a^ |
| **Sparrow** | 10.7±2.59^cd^ | 2.6±1.13^abc^ | 217.0±109.00^bcd^ | 11.0±6.35^de^ | 39.4±14.38^c^ | 8.6±0.71^bcd^ | 9.6±4.67^ab^ | 79.7±27.15^a^ | 10.2±0.56^a^ | 24.9±3.18^a^ | 4.5±0.73^a^ |
| **Surprise** | 10.4±0.59^cde^ | 2.5±0.32^abc^ | 349.0±35.60^a^ | 22.5±2.79^c^ | 33.3±15.63^c^ | 12.2±1.08^a^ | 9.3±0.40^ab^ | 38.6±17.58^a^ | 9.6±0.70^a^ | 23.5±4.37^a^ | 3.3±0.11^a^ |
| **Tomboy** | 18.0±0.65^a^ | 2.4±0.07^abc^ | 305.0±3.82^abc^ | 16.8±0.71^cd^ | 45.5±1.40^c^ | 8.2±0.93^cd^ | 8.4±0.74^ab^ | 29.0±11.41^a^ | 9.0±1.23^a^ | 20.4±3.11^a^ | 3.7±0.48^a^ |
| **Sparks 147** | 6.0±0.59^de^ | 1.7±0.23^bc^ | 146.0±8.37^de^ | 2.6±0.14^f^ | 36.3±0.77^c^ | 7.7±0.98^cd^ | 8.9±0.76^ab^ | 37.0±24.27^a^ | 10.0±0.90^a^ | 25.7±1.67^a^ | 3.6±0.45^a^ |
| **Average** | 11.1±1.27 | 2.5±0.44 | 233.5±22.27 | 15.2±1.82 | 61.4±11.76 | 9.2±0.94 | 9.5±1.39 | 47.1±25.39 | 9.7±1.22 | 26.4±6.30 | 3.6±0.51 |
| **P-value** | **0.00*** | **0.00*** | **0.00*** | **0.00*** | **0.00*** | **0.00*** | **0.01*** | **0.15** | **0.89** | **0.68** | **0.25** |

*There is a significant difference among some cultivars.

^abcdef^  Means in the same column with the same letter are not significantly different.
